# *Solanum nigrum* Protects against Hepatic Fibrosis via Suppression of Hyperglycemia in High-Fat/Ethanol Diet-Induced Rats

**DOI:** 10.3390/molecules21030269

**Published:** 2016-02-25

**Authors:** Cheng-Jeng Tai, Chen-Yen Choong, Yeu-Ching Shi, Yu-Chun Lin, Chia-Woei Wang, Bao-Hong Lee, Chen-Jei Tai

**Affiliations:** 1 Division of Hematology and Oncology, Department of Internal Medicine, Taipei Medicine University Hospital, Taipei 11031, Taiwan; cjtai@tmu.edu.tw; 2 Department of Internal Medicine, School of Medicine, College of Medicine, Taipei Medical University, Taipei 11031, Taiwan; 3 Department of Obstetrics and Gynecology, School of Medicine, College of Medicine, Taipei Medical University, Taipei 11031, Taiwan; chenyen_318@hotmail.com (C.-Y.C.); cwwang@ms4.hinet.net (C.-W.W.); 4 Taiwan Indigena Botanica Co., Ltd., Taipei 11458, Taiwan; jasmineycs@yahoo.com.tw; 5 Graduate Institute of Medical Sciences, Taipei Medical University, Taipei 11031, Taiwan; doclin10@gmail.com; 6 Department of Obstetrics and Gynecology, Taipei Medical University Hospital, Taipei 11031, Taiwan; 7 Department of Chinese Medicine, Taipei Medical University Hospital, Taipei 11031, Taiwan; 8 Traditional Herbal Medicine Research Center, Taipei Medical University Hospital, Taipei 11031, Taiwan

**Keywords:** advanced glycation end products (AGEs), hyperglycemia, *Solanum nigrum*, hyperlipidemia, hepatic fibrosis

## Abstract

Background: Advanced glycation end products (AGEs) signal through the receptor for AGE (RAGE), which can lead to hepatic fibrosis in hyperglycemia and hyperlipidemia. We investigated the inhibitory effect of aqueous extracts from *Solanum nigrum* (AESN) on AGEs-induced RAGE signaling and activation of hepatic stellate cells (HSCs) and hyperglycemia induced by high-fat diet with ethanol. Methods: An animal model was used to evaluate the anti-hepatic fibrosis activity of AESN in rats fed a high-fat diet (HFD; 30%) with ethanol (10%). Male Wistar rats (4 weeks of age) were randomly divided into four groups (*n* = 6): (1) control (basal diet); (2) HFD (30%) + ethanol (10%) (HFD/ethanol); (3) HFD/ethanol + AESN (100 mg/kg, oral administration); and (4) HFD/ethanol + pioglitazone (10 mg/kg, oral administration) and treated with HFD for 6 months in the presence or absence of 10% ethanol in dietary water. Results: We found that AESN improved insulin resistance and hyperinsulinemia, and downregulated lipogenesis via regulation of the peroxisome proliferator-activated receptor α (PPARα), PPARγ co-activator (PGC-1α), carbohydrate response element-binding protein (ChREBP), acetyl-CoA carboxylase (ACC), and fatty acid synthase (FAS) mRNA levels in the liver of HFD/ethanol-treated rats. In turn, AESN may delay and inhibit the progression of hepatic fibrosis, including α-smooth muscle actin (α-SMA) inhibition and MMP-2 production. Conclusions: These results suggest that AESN may be further explored as a novel anti-fibrotic strategy for the prevention of liver disease.

## 1. Introduction

Nonalcoholic fatty liver disease (NAFLD) disorders range from simple fatty liver (steatosis without liver injury) to nonalcoholic steatohepatitis (NASH) and fibrosis/cirrhosis, which have been reported in a recent study [1]. The pathogenesis and progression of hepatic fibrosis were described by “two-hit theory” in NAFLD and NASH. In this theory, the first hit consists of fatty acids/triglycerides accumulation in the liver (NAFLD and NASH), while the second hit involves oxidative stress, mitochondrial dysfunction, and inflammation, which ultimately cause hepatic fibrosis by ethanol or inflammation [2]. In the current study, we firstly induced hyperlipidemia and hyperglycemia for NASH development, and subsequently induced hepatic fibrosis by ethanol administration as an animal model for evaluation of *Solanum nigrum* against liver damage by blocking “two-hit theory”.

Hyperglycemia and hyperlipidemia are common causes of chronic liver disease, which is strongly associated with insulin resistance, leading to NASH and hepatic fibrosis [3,4,5]. Hepatic stellate cells (HSCs) are fat-storing cells activated upon liver injury. Importantly, HSC activation can lead to a myofibroblastic phenotype, thereby contributing to fibrotic processes. NASH is a common but often silent chronic liver disease that clinically and histologically resembles alcoholic liver disease that occurs in people who drink little or no alcohol [6,7]. NAFLD is defined as elevation of hepatic bioindex, lipids accumulation in the liver of individuals who do not consume significant amounts of alcohol [8]. The amount of ethanol consumed determines its role in the nutritional balance; therefore, alcohol abuse leads to alcoholic fatty liver disease [9]. However, the effect of light alcohol consumption (approximately 140 g/week) on NAFLD has not been well documented [10].

Fast-food consumption is associated with weight gain, insulin resistance, and liver damage. Insulin resistance plays an important role in the development of type 2 diabetes mellitus, which almost always involves hepatic fibrosis. Recently, the suppression of diabetes and the metabolic syndrome were shown to protect liver against hepatic fibrosis) [11]. In diabetic patients caused by obesity, there is a positive correlation between high concentrations of advanced glycation end products (AGEs) in the blood and hyperglycemia [4]. Further, hyperglycemia facilitates the formation of AGEs in type-2 diabetes. AGEs signals through receptors for AGEs (RAGE), resulting in hepatic fibrosis via hyperglycemia [5]. There is a positive correlation between a high methylglyoxal concentration in the blood and hyperglycemia in diabetic patients, [4]. Methylglyoxal is a highly reactive dicarbonyl compound and metabolic product of glucose, and several lines of clinical evidence suggest that methylglyoxal reacts with proteins, resulting in the irreversible formation of AGEs in patients with hyperglycemia and diabetes [3].

*Solanum* *nigrum* (SN) is a kind of herb that widely used as an elemental ingredient in traditional Chinese medicine formulas for cancer therapy. Recently, we have found that aqueous extract of *Solanum* *nigrum* (AESN) could show anti-proliferation ability in various cancer cells [12,13,14,15]. In addition to suppressing cancer cells, *Solanum* *nigrum* has also been reported to protect liver damage and hepatic fibrosis caused by chemicals [16,17]. However, the regulation of *Solanum* *nigrum* on HSCs activation is not clear. We considered that AESN could improve metabolic syndrome against activation of HSCs. In this study, the ability of AESN to attenuate liver damage induced by high-fat/ethanol diet remains unclear. Thus, the aim of the study was to investigate the regulatory mechanism of AESN during liver damage and HSCs activation *in vivo* and *in vivo*.

## 2. Results

### 2.1. AESN Inhibits Hepatic Fibrosis

**Figure 1:**
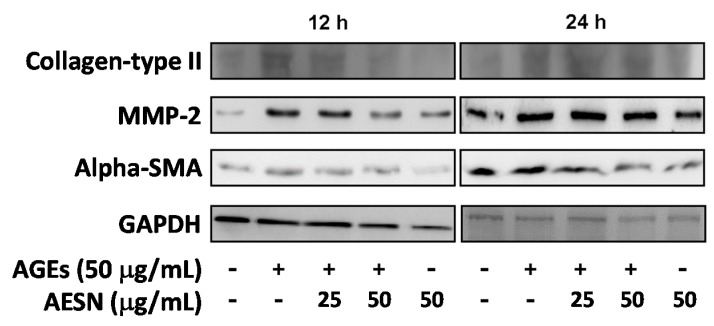
The inhibition of AESN on activation of HSC-t6 cells caused by AGEs induction.

We previously used high-fructose diet to induce hepatic damage caused by NAFLD in rats [18]. In addition, we also confirmed that HSCs were activation by AGEs induction [5,11]. In this study, we used AGEs to induce the HSCs-t6 cells activation, and we found that AESN (10 μM) treatment could inhibit the enhanced MMP-2 and α-SMA expression caused by AGEs (Figure 1).

### 2.2. Anti-Diabetic Activity of AESN

**Figure 2:**
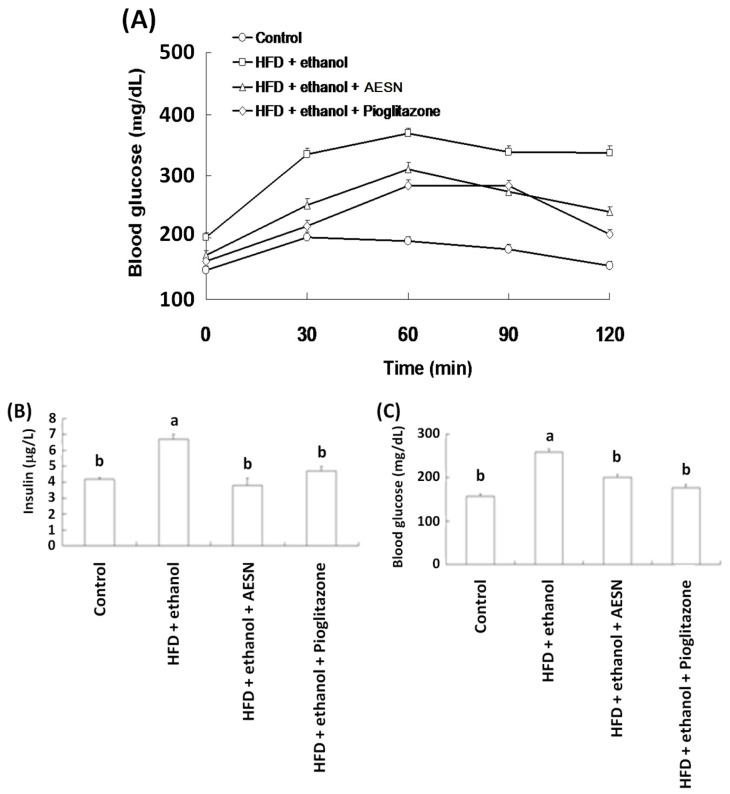
The effects of AESN on (**A**) OGTT; (**B**) insulin; and (**C**) blood glucose in HFD/ethanol-induced rats. Data were shown as mean ± SD (*n* = 6). Significantly difference was shown as various letters (a, b, c) (*p* < 0.05).

**Figure 3:**
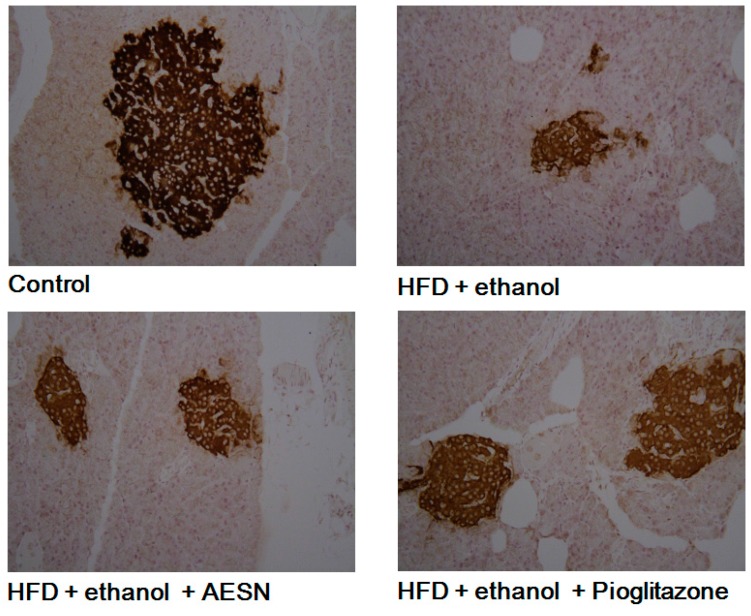
The results of IHC stain for the level of pancreatic insulin.

The effects of AESN and pioglitazone on the oral glucose tolerance test (OGTT) and the insulin tolerance test (ITT) were evaluated after HFD/ethanol treatments. The OGTT is an index that evaluates blood glucose changes after glucose administration *in vivo*. Pioglitazone is a clinical PPAR agonist and has been used to prevent diabetes. As shown in Figure 2A, HFD/ethanol treatment significantly increased hyperglycemia after oral glucose administration when compared with the control group. After sacrificing the rats, serum insulin and blood glucose levels were measured. As shown in Figure 2B,C, AESN markedly lowered serum insulin and blood glucose in HFD/ethanol-treated rats, suggesting that AESN markedly attenuated insulin resistance. Pancreatic insulin expression was evaluated via IHC staining. HFD/ethanol treatments suppressed pancreatic insulin levels in rats (Figure 3). AESN did not recover insulin expression; however pioglitzone clearly elevated insulin expression.

### 2.3. AESN Protects against Liver Damage Induced by HFD/Ethanol Treatment

**Figure 4:**
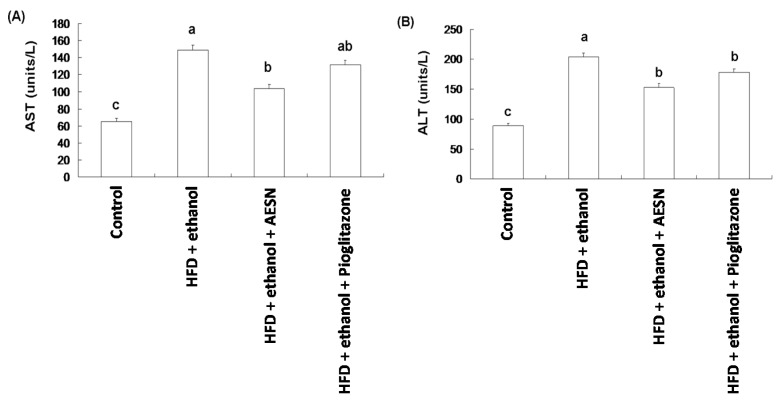
The hepatoprotection of AESN against liver damage index (**A**) AST and (**B**) ALT induced by HFD/ethanol. Data were shown as mean ± SD (*n* = 6). Significantly difference was shown as various letters (a, ab, b, c) (*p* < 0.05).

**Figure 5:**
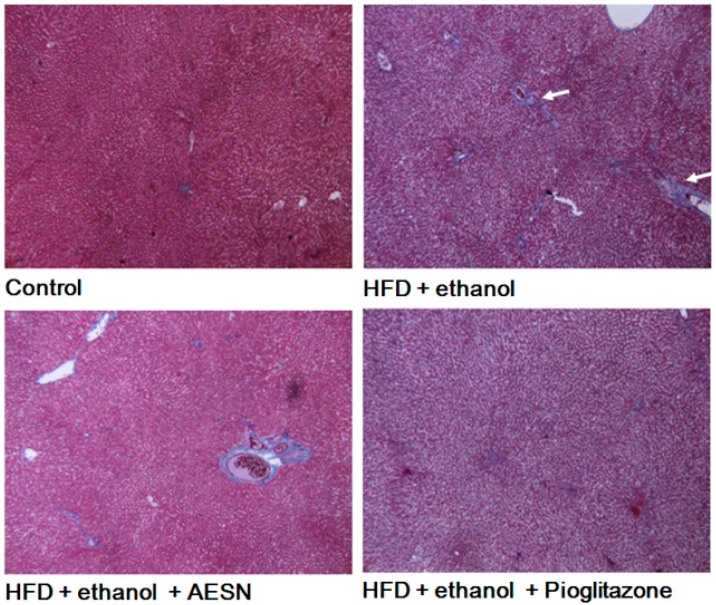
The results of AESN against hepatic fibrosis by Masson’s Trichrome stain in the liver of HFD/ethanol-induced rats. Data were shown as mean ± SD (*n* = 6).

**Figure 6:**
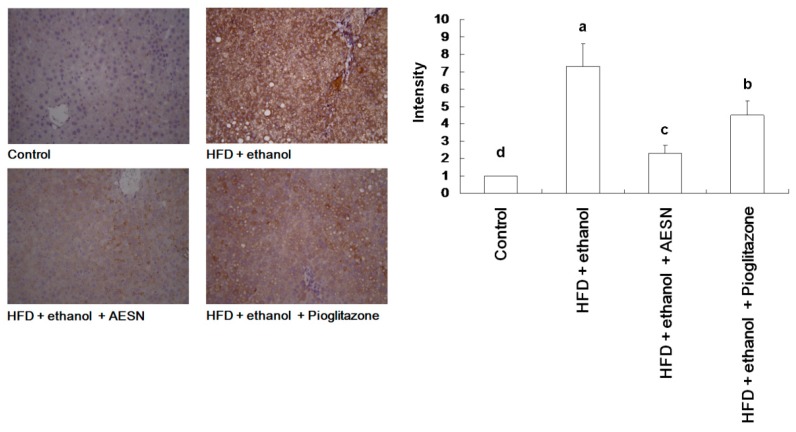
The results of IHC stain on the level of alpha-SMA in the liver of HFD/ethanol-induced rats. Data were shown as mean ± SD (*n* = 6). Significantly difference was shown as various letters (a, b, c, d) (*p* < 0.05).

In clinical assessments, serum AST and ALT levels are the indexes of hepatic function. These indicators are likely released in the serum from the liver during hepatic cell death. Serum AST and ALT levels were elevated in rats administered HFD/ethanol when compared with the control group, indicating that HFD/ethanol treatments caused hepatic toxicity (Figure 4). Elevations in the serum AST and ALT levels were significantly reduced in the AESN and pioglitazone treated rats. Liver damage was assessed by histopathological staining of collagen accumulation. As shown in Figure 5, HFD/ethanol administration markedly elevated collagen accumulation and fatty degeneration. However, AESN and pioglitzone administration effectively improved hepatic fibrosis induced by HFD/ethanol. A second indicator of hepatic fibrosis, α-SMA, which is expressed in the liver of HFD/ethanol treated-rats, was also investigated by IHC stain. Our results indicated that HFD/ethanol treatment significantly increased the production of α-SMA while AESN and pioglitazone attenuated this situation (Figure 6).

### 2.4. AESN Improves Fatty Liver

**Figure 7:**
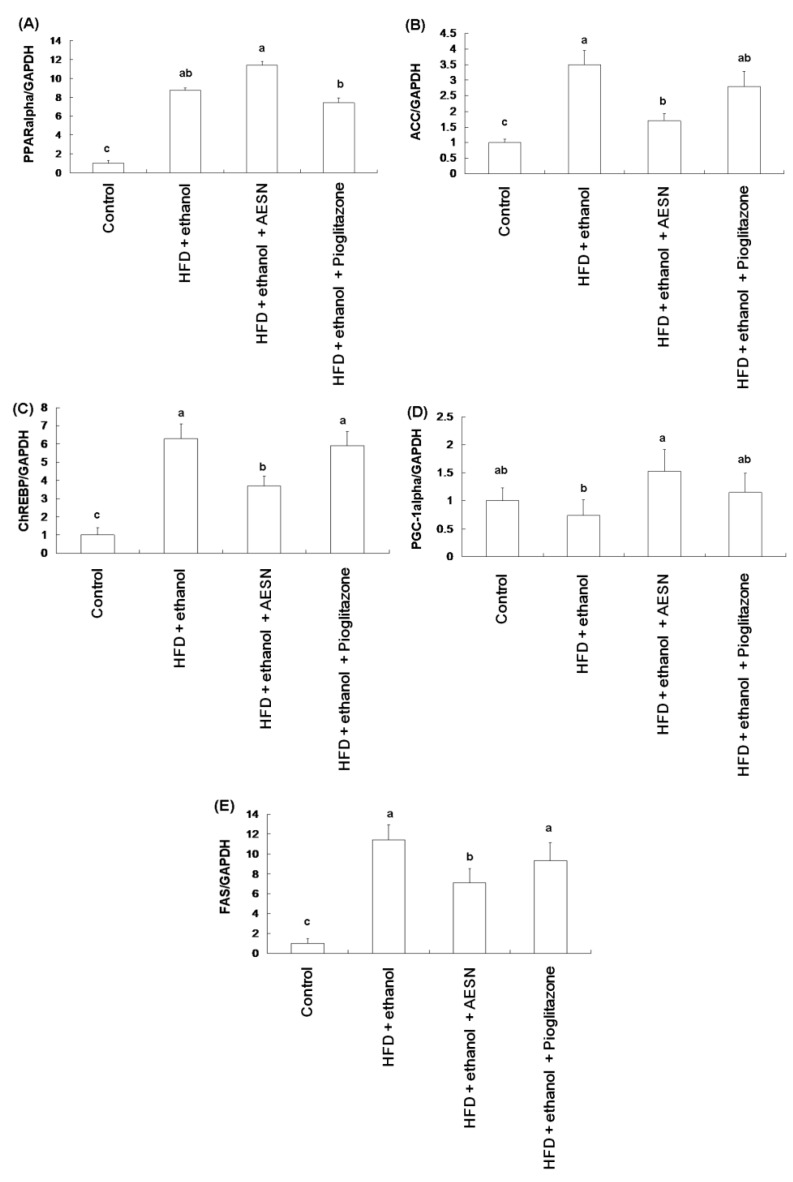
The regulation of AESN on hepatic (**A**) PPARalpha; (**B**) ACC; (**C**) ChREBP; (**D**) PGC-1alpha; and (**E**) FAS mRNA levels in HFD/ethanol-induced rats. Data were shown as mean ± SD (*n* = 6). Significantly difference was shown as various letters (a, ab, b, c) (*p* < 0.05).

Our results demonstrated that the HFD/ethanol treatment elevated serum levels of total cholesterol (TC), triglycerides (TG), and free fatty acids (FFA), but decreased serum high density lipoprotein cholesterol (HDL-C) level (Table 1). AESN and pioglitazone inhibited the elevation of serum TC, TG, and FFA of rats induced by HFD/ethanol, but had no effect on HDL-C level in AESN and pioglitazone treated rats. Moreover, we investigated the expression of hepatic genes associated with lipogenesis and β-oxidation. As shown in Figure 7, HFD/ethanol treatment markedly elevated the mRNA levels of PPARα, acetyl-CoA carboxylase (ACC), carbohydrate response element-binding protein (ChREBP), and fatty acid synthase (FAS) while AESN suppressed these genes expression, which showing a greater effect than pioglitazone. Moreover, AESN significantly increased the mRNA level of PPARγ co-activator (PGC-1α) as compared to the HFD/ethanol treatment group. These results suggested that AESN could improve hyperglycemia and hyperlipidemia to protect liver against from damage, thereby arresting hepatic fibrosis.

### 2.5. AESN Inhibits RAGE in HFD/Ethanol Treated-Rats

**Figure 8:**
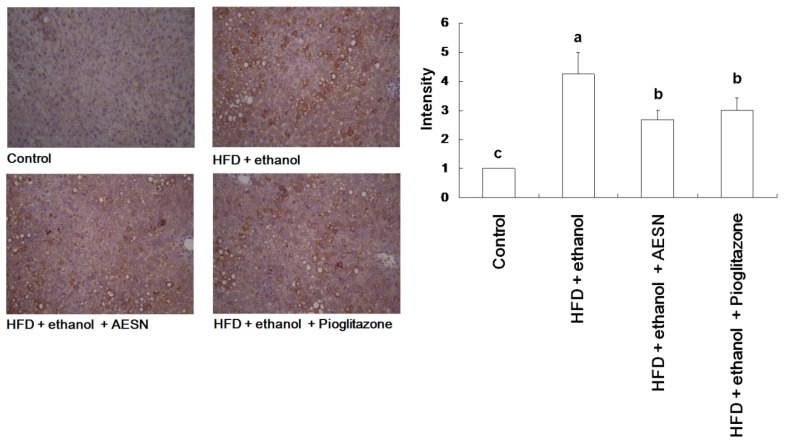
The results of IHC stain for the level of AGEs in liver of HFD/ethanol-induced rats. Data were shown as mean ± SD (*n* = 6). Significantly difference was shown as various letters (a, b, c) (*p* < 0.05). The brown appearance is target AGEs stain.

**Figure 9:**
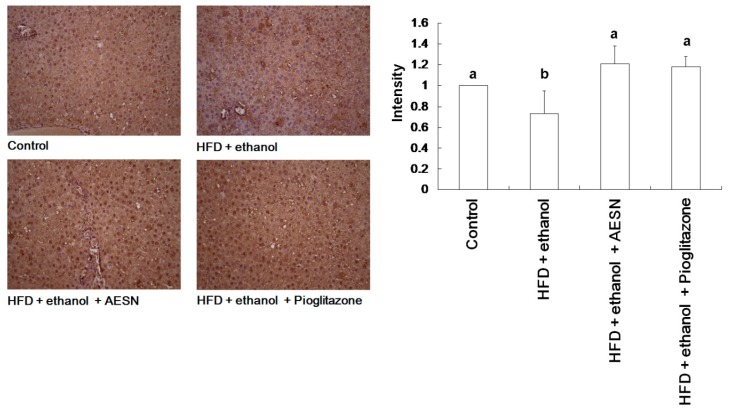
The results of IHC stain for the level of Nrf2 in liver of HFD/ethanol-induced rats. Data were shown as mean ± SD (*n* = 6). Significantly difference was shown as various letters (a, b) (*p* < 0.05). The brown appearance is target Nrf2 stain.

The metabolic syndrome is associated with obesity, hypertension, dyslipidemia, and insulin resistance [19]. In addition, AGEs accumulation is observed in high-fat diet-induced mice [20]. Thus, AGEs are considered a biomarker of these diseases. We found that HFD/ethanol treatment significantly elevated AGE accumulation in the liver; however, AESN effectively attenuated AGEs formation and deposition (Figure 8). We showed that nuclear factor-erythroid 2-related factor 2 (Nrf2) activation could attenuate the toxic effects of methylglyoxal by inducing glyoxalase 1 expression, which metabolizes methylglyoxal to D-lactic acid [21,22]. Some antioxidants have been reported to attenuate oxidative damage by activating Nrf2 [23]. Therefore, we investigated the effect of AESN on hepatic Nrf2 expression by IHC stain. As shown in Figure 9, AESN enhanced Nrf2 expression when compared with the HFD/ethanol treatment. These results indicate that AESN may elevate Nrf2 to attenuate AGE formation, thereby preventing hepatic fibrosis.

## 3. Discussion

In this study, the rats were treated with HFD over 6 months to induce hyperlipidemia and hyperglycemia, and followed by oral ethanol administration to induce HSCs activation for 2 months. During the ethanol treatment, AESN was simultaneously administered. AESN was found to preventhyperglycemia, hyperlipidemia, and hepatic damage. We found that AESN attenuated AGEs formation and suppressed AGEs accumulation in the liver), and elevated Nrf2 expression in the liver of rats, suggesting that AESN could avoid oxidative stress. Moreover, AESN also promoted lipid metabolism and avoided NAFLD, which were considered to be the regulatory effects of AESN for hepatic fibrosis. In addition, we found that AESN inhibited AGEs-induced HSCs activation and the expression of hepatic fibrogenetic biomarkers, including MMP-2 and α-SMA, independent of RAGE activation. Further, AESN protected liver from damage by preventing the hepatic fibrosis through the suppression of α-SMA level in the liver of HFD/ethanol-treated rats. Additionally, AESN could improve indexes associated with liver damage, including AST and ALT.

AESN also elevated Nrf2 expression in the liver of HFD/ethanol-induced rats. Glyoxalase-1 catalyzes the conversion of methylglyoxal to S-d-lactoylglutathione, whereas glyoxalase-2 hydrolyzes the glutathione thiolester to d-lactic acid and glutathione, depending on Nrf2 activation. Taken together, AESN may downregulate HSCs activation by inhibiting AGE-associated signaling pathways involved in anti-glycation and Nrf2 activity.

The anti-hyperglycemia effect of *Solanum* *nigrum* fruits has been investigated in a previous study [24,25]. However, the effect of AESN on hyperlipidemia remains unknown. We observed that AESN markedly protected against insulin resistance and increased blood glucose, independent of insulin expression. We found that AESN regulated the expression of several genes associated with lipogenesis. The transcription factor PPARα and the co-activator PGC-1α play a key role in fatty acid oxidation [18]. ChREBP is a major transcription factor regulating fatty acid synthesis in the liver, and controls approximately half of the steps involved in hepatic lipogenesis by regulating the transcription of glycolytic and lipogenic genes, including ACC and FAS [26,27]. Moreover, high-fat feeding induces the PGC-1 family of co-activators, including PGC-1α and PGC-1β, which coordinately enhances ChREBP-mediated upregulation of lipogenic enzymes [28,29]. We found that AESN elevated PPARα and PGC-1α mRNA levels in HFD/ethanol-induced rats, and inhibited the decrease in ACC, FAS, and ChREBP mRNA levels (Figure 6), thereby improving hyerlipidemia (Table 1) and suppress HSCs activation and hepatic fibrosis.

Solanine is an active compound in *Solanum* *nigrum*, which has been reported to inhibit cancer cells and anti-inflammation [30,31]. In addition, the biofunctions of solanine has also been described [32], we found that AESN showed the anti-hepatic fibrosis activity in current study. However, the inhibition of HSCs activity by solanine should be investigated by further study. HFD can induce AGEs and methglyoxal accumulation. Recently, several foods and drinks, including coffee, honey, and cake, were shown to contain high levels of AGEs [33,34,35]. Taken together, AESN could potentially be supplied in food supplements, or developed as functional foods, to minimize the development of diabetes, hyperlipidemia, and hepatic fibrosis.

## 4. Experimental Section

### 4.1. Chemicals

*Solanum* *nigrum* was purchased from a local market in Tainan, Taiwan. The extracts were prepared with hot water for 30 min extraction, and the extracts were vacuum-concentrated and freeze dried. Fetal bovine serum (FBS), Waymouth’s medium, sodium bicarbonate, penicillin, and streptomycin were purchased from HyClone Laboratories (Logan, UT, USA). Anti-AGE antibodies were purchased from Abcam (Cambridge, MA, USA). Anti-α-smooth muscle actin (α-SMA) antibody, anti-type II collagen antibody, and anti-matrix metalloproteinase-2 (MMP-2) antibody were purchased from Santa Cruz Biotechnology (Santa Cruz, CA, USA). The Bio-Rad protein assay dye was purchased from Bio-Rad Laboratories (Hercules, CA, USA).

### 4.2. Animal Treatments

Male Wistar rats (4 weeks of age) were obtained from the National Laboratory Animal Breeding and Research Center (Taipei, Taiwan). Animals were acclimatized for 1 week before use. Animals were provided with food and water *ad libitum* and kept under a 12 h light/dark cycle with a relative humidity of 60% and a temperature of 25 °C. The protocol complied with guidelines described in the Taiwanese Animal Protection Law, as amended on 17 January 2001 (Hua-Zong-(1)-Yi-Tzi-9000007530, Council of Agriculture, Executive Yuan, Taiwan). Rats were randomly divided into 4 treatment groups (*n* = 6) and treated for 6 months with HFD induction in the presence or absence of 10% ethanol in dietary water for 2 months. The groups were: (1) control (basal diet); (2) HFD (30%) + ethanol (10%) (HFD/ethanol); (3) HFD/ethanol + AESN (100 mg/kg, oral administration); and (4) HFD/ethanol + pioglitazone (10 mg/kg, oral administration).

### 4.3. Oral Glucose Tolerance Test (OGTT)

The OGTT was performed after overnight fasting. Glucose was orally administered (2 g/kg body weight), and blood glucose levels were determined using a glucose assay kit (BioAssay Systems, Hayward, CA, USA) [4].

### 4.4. Assays for Serum Insulin, Aspartate Transaminase (AST), and Alanine Transaminase (ALT)

Serum insulin levels were assayed using an enzyme-linked immunosorbent assay (ELISA) kit (Mercodia, Winston Salem, NC, USA). AST and ALT levels were assayed using commercial kits from Randox Laboratories Ltd. (Crumlin, Co., Antrim, UK).

### 4.5. RNA Preparation and Real-Time PCR

Total RNA was isolated using Trizol (Life Technologies, Carlsbad, CA, USA) according to the manufacturer’s instructions. cDNA from 3 μg of RNA was generated using SuperScript III First-Strand Synthesis System for RT-PCR (Life Technologies) according to the manufacturer’s instructions. The reverse-transcription product was diluted in water, and a volume corresponding to 30 ng of original RNA was used for real-time PCR. Real-time PCR amplification and detection were performed using the SYBR Green qPCR SuperMix-uracil DNA glycosylase (UDG) with ROX (Life Technologies) in a fluorescence thermal cycler (StepOne Real-Time PCR System, Life Technologies) according to the manufacturer’s protocol. Gene expression was normalized using GAPDH as a reference gene.

### 4.6. Hepatic Fibrosis Stain

Masson’s Trichrome was used to differentiate collagen from other fibers in the histopathological analysis.

### 4.7. Immunohistochemistry (IHC) Stain

Tissue sections were incubated with 3% H_2_O_2_ for 20 min to quench endogenous peroxidase activity. After rinsing twice with PBS, the sections were incubated with skimmed milk (5%) for 1 h, and the primary monoclonal antibody for 12 h at 4 °C. After washing with PBS, the sections were incubated with the secondary antibody (1:200) in PBS for 1 h. After the sections were rinsed twice with PBS, immunoreactions were visualized by incubation with 3,3′-diaminobenzidine tetrahydrochloride for 10 min. The sections were counterstained with hematoxylin and eosin.

### 4.8. HSCs-t6 Cell Culture

The HSCs-t6 cell line was cultured in Waymouth’s medium with 10% FBS. Cells were treated with AGEs for 24 h, with or without AESN treatment, for α-SMA, type II collagen, and MMP-2 assays by Western blot.

### 4.9. Western Blot

Proteins were detected by 10% sodium dodecyl sulfate-polyacrylamide gel electrophoresis (SDS-PAGE) and then transferred to a nitrocellulose (NC) membrane. The NC membrane was incubated with primary and secondary antibodies, and proteins were detected using an enhanced chemiluminescent (ECL) reagent (Millipore, Billerica, MA, USA).

### 4.10. Statistical Analysis

Experimental results were analyzed in triplicate and expressed as the mean ± standard deviation (SD). The results were subjected to one-way analysis of variance (ANOVA) and Duncan’s multiple range tests. A *p*-value less than 0.05 was considered significant.

## 5. Conclusions

Our results suggest that AESN may be further explored as a novel anti-fibrotic strategy for the prevention of liver disease.

## Figures and Tables

**Table:** 

	Mean Score	SD
Control	0	0
HFD + Ethanol	3.26	0.45
HFD + Ethanol + AESN	2.73	0.38
HFD + Ethanol + Pioglitazone	2.15	0.52

**Table 1:** The effect of AESN on serum biochemical values.

Groups	TC (mg/dL)	TG (mg/dL)	HDL-C (mg/dL)	FFA (mg/dL)
Control	83.5 ± 13.0 ^c^	38.7 ± 7.8 ^d^	27.6 ± 3.7 ^a^	0.76 ± 0.04 ^c^
HFD + Ethanol	187.6 ± 17.2 ^a^	89.1 ± 5.7 ^a^	14.5 ± 1.3 ^b^	4.63 ± 0.16 ^a^
HFD + Ethanol + AESN	137.3 ± 14.1 ^b^	57.3 ± 7.8 ^c^	13.3 ± 2.2 ^b^	2.45 ± 0.15 ^b^
HFD + Ethanol + Pioglitazone	164.2 ± 15.2 ^a,b^	69.0 ± 8.4 ^b^	15.7 ± 3.2 ^b^	3.41 ± 0.11 ^a^

Data were shown as mean ± SD (*n* = 6). Significantly difference was shown as various letters (a, b, c, d) (*p* < 0.05). dL= 100 mL.

## Abbreviations

ACC: acetyl-CoA carboxylase
AESN: aqueous extracts from *Solanum* *nigrum*
AGEs: Advanced glycation end products
ChREBP: carbohydrate response element-binding protein
FAS: fatty acid synthase
FBS: fetal bovine serum
FFA: free fatty acids
HASH: nonalcoholic steatohepatitis
HDL-C: high density lipoprotein cholesterol
HFD: high-fat diet
HSCs: hepatic stellate cells
ITT: insulin tolerance test
Nrf2: nuclear factor-erythroid 2-related factor 2
OGTT: oral glucose tolerance test
PGC-1α: PPARγ co-activator (PGC-1)
PPARα: peroxisome proliferator-activated receptor α
RAGE: Receptor for AGEs
TC: cholesterol
TG: triglycerides

## References

[1] Salt W.B. (2004). Nonalcoholic fatty liver disease (NAFLD): A comprehensive review. J. Insur. Med..

[2] Lalor P.F., Faint J., Aarbodem Y., Hubscher S.G., Adams D.H. (2007). The role of cytokines and chemokines in the development of steatohepatitis. Semin. Liver Dis..

[3] Hsu W.H., Chen T.H., Lee B.H., Hsu Y.W., Pan T.M. (2014). Monascin and ankaflavin act as natural AMPK activators with PPARalpha agonist activity to down-regulate nonalcoholic steatohepatitis in high-fat diet-fed C57BL/6 mice. Food Chem. Toxicol..

[4] Hsu W.H., Lee B.H., Chang Y.Y., Hsu Y.W., Pan T.M. (2013). A novel natural Nrf2 activator with PPARgamma-agonist (monascin) attenuates the toxicity of methylglyoxal and hyperglycemia. Appl. Toxicol. Pharmacol..

[5] Hsu W.H., Lee B.H., Hsu Y.W., Pan T.M. (2013). Peroxisome proliferator-activated receptor-gamma activators monascin and rosiglitazone attenuate carboxymethyllysine-induced fibrosis in hepatic stellate cells through regulating the oxidative stress pathway but independent of the receptor for advanced glycation end products signaling. J. Agric. Food Chem..

[6] Bacon B.R., Farahvash M.J., Janney C.G., Neuschwander-Tetri B.A. (1994). Nonalcoholic steatohepatitis: An expanded clinical entity. Gastroenterology.

[7] Ludwig J., Viggiano T.R., McGill D.B., Oh B.J. (1980). Nonalcoholic steatohepatitis: Mayo Clinic experiences with a hitherto unnamed disease. Mayo Clin. Proc..

[8] Dam-Larsen S., Franzmann M., Andersen I.B., Christoffersen P., Jensen L.B., Sorensen T.I., Becker U., Bendtsen F. (2004). Long term prognosis of fatty liver: Risk of chronic liver disease and death. Gut.

[9] Zhong W., Zhao Y., Tang Y. (2012). Chronic alcohol exposure stimulates adipose tissue lipolysis in mice: Role of reverse triglyceride transport in the pathogenesis of alcoholic steatosis. Am. J. Pathol..

[10] Hamaguchi M., Kojima T., Ohbora A. (2012). Protective effect of alcohol consumption for fatty liver but not metabolic syndrome. World J. Gastroenterol..

[11] Lee B.H., Hsu W.H., Hsu Y.W., Pan T.M. (2013). Suppression of dimerumic acid on hepatic fibrosis caused from carboxymethyl-lysine (CML) by attenuating oxidative stress depends on Nrf2 activation in hepatic stellate cells (HSCs). Food Chem. Toxicol..

[12] Wang C.K., Lin Y.F., Tai C.J., Wang C.W., Chang Y.J., Choong C.Y., Lin C.S., Tai C.J., Chang C.C. (2015). Integrated treatment of aqueous extract of *Solanum nigrum*-potentiated cisplatin- and doxorubicin-induced cytotoxicity in human hepatocellular carcinoma cells. Evid. Based Complement. Altern. Med..

[13] Wang C.W., Chen C.L., Wang C.K., Chang Y.J., Jian J.Y., Lin C.S., Tai C.J., Tai C.J. (2015). Cisplatin-, doxorubicin- and docetaxel-induced cell death promoted by the aqueous extract of *Solanum nigrum* in human ovarian carcinoma cells. Integr. Cancer Ther..

[14] Tai C.J., Wang C.K., Chang Y.J., Lin C.S., Tai C.J. (2012). Aqueous extract of *Solanum nigrum* leaf activates autophagic cell death and enhances docetaxel-induced cytotoxicity in human endometrial carcinoma cells. Evid. Based Complement. Altern. Med..

[15] Tai C.J., Wang C.K., Tai C.J., Lin Y.F., Lin C.S., Jian J.Y., Chang Y.J., Chang C.C. (2013). Aqueous extract of *Solanum nigrum* leaves induces autophage and enhances cytotoxicity of cisplatin, doxorubicin, docetaxel, and 5-fluorouracil in human colorectal carcinoma cells. Evid. Based Complement. Altern. Med..

[16] Lin H.M., Tseng H.C., Wang C.J., Lin J.J., Lo C.W., Chou F.P. (2008). Hepatoprotective effects of *Solanum nigrum* Linn extract against CCl(4)-induced oxidative damage in rats. Chem. Biol. Interact..

[17] Hsieh C.C., Fang H.L., Lina W.C. (2008). Inhibitory effect of *Solanum nigrum* on thioacetamide-induced liver fibrosis in mice. J. Ethnopharmacol..

[18] Lee B.H., Hsu W.H., Huang T., Chang Y.Y., Hsu Y.W., Pan T.M. (2013). Monascin improves diabetes and dyslipidemia by regulating PPARgamma and inhibiting lipogenesis in fructose-rich diet-induced C57BL/6 mice. Food Funct..

[19] Hudson B.I., Dong C., Gardener H. (2014). Serum levels of soluble receptor for advanced glycation end-products and metabolic syndrome: The Northern Manhattan study. Metabolism.

[20] Leuner B., Max M., Thamm K. (2012). RAGE influences obesity in mice. Effects of the presence of RAGE on weight gain, AGE accumulation, and insulin levels in mice on a high fat diet. Z. Gerontol. Geriatr..

[21] Lee B.H., Hsu W.H., Chang Y.Y., Kuo H.F., Hsu Y.W., Pan T.M. (2012). Ankaflavin: A natural novel PPARγ agonist upregulates Nrf2 to attenuate methylglyoxal-induced diabetes *in vivo*. Free Radic. Biol. Med..

[22] Vander-Jagt D., Hunsaker L. (2003). Methylglyoxal metabolism and diabetic complications: Roles of aldose reductase, glyxalase-I, betaine aldehyde dehydrogenase and oxoaldehyde dehydrogenase. Chem. Biol. Int..

[23] Weng C.J., Chen M.J., Yeh C.T., Yen G.C. (2011). Hepatoprotection of quercetin against oxidative stress by induction of metallothionein expression through activating MAPK and PI3K pathways and enhancing Nrf2 DNA-binding activity. New Biotechnol..

[24] Sohrabipour S., Kharazmi F., Soltani N., Kamalinejad M. (2013). Effect of the administration of *Solanum nigrum* fruit on blood glucose, lipid profiles, and sensitivity of the vascular mesenteric bed to phenylephrine in streptozotocin-induced diabetic rats. Med. Sci. Monit. Basic Res..

[25] Sohrabipour S., Kharazmi F., Soltani N., Kamalinejad M. (2014). Biphasic effect of *Solanum nigrum* fruit aqueous extract on vascular mesenteric beds in non-diabetic and streptozotocin-induced diabetic rats. Pharmacogn. Res..

[26] Uyeda K., Repa J.J. (2006). Carbohydrate response element binding protein, ChREBP, a transcription factor coupling hepatic glucose utilization and lipid synthesis. Cell Metab..

[27] Iizuka K., Horikawa Y. (2008). ChREBP: A glucose-activated transcription factor involved in the development of metabolic syndrome. Endocr. J..

[28] Repa J.J., Liang G., Ou J. (2000). Regulation of mouse sterol regulatory element-binding protein-1c gene (SREBP-1c) by oxysterol receptors, LXRalpha and LXRbeta. Genes Dev..

[29] Lin J., Yang R., Tarr P.T. (2005). Hyperlipidemic effects of dietary saturated fats mediated through PGC-1beta coactivation of SREBP. Cell.

[30] Shen K.H., Liao A.C., Hung J.H., Lee W.J., Hu K.C., Lin P.T., Liao R.F., Chen P.S. (2014). Alpha-Solanine inhibits invasion of human prostate cancer cells by suppressing epithelial-mesenchymal transition and MMPs expression. Molecules.

[31] Kang H., Jeong H.D., Choi H.Y. (2011). The chloroform fraction of *Solanum nigrum* suppresses nitric oxide and tumor necrosis factor-alpha in LPS-stimulated mouse peritoneal macrophages through inhibition of p38, JNK and ERK1/2. Am. J. Chin. Med..

[32] Friedman M. (2004). Analysis of biologically active compounds in potatoes (*Solanum tuberosum*), tomatoes (*Lycopersicon esculentum*), and jimson weed (*Datura stramonium*) seeds. J. Chromatogr. A.

[33] Wang J., Chang T. (2010). Methylglyoxal content in drinking coffee as a cytotoxic factor. J. Food Sci..

[34] Adams C.J., Manley-Harris M., Molan P.C. (2009). The origin of methylglyoxal in New Zealand manuka (*Leptospermum scoparium*) honey. Carbohydr. Res..

[35] Arrbias-Lorenzo G., Morales F.J. (2010). Analysis, distribution, and dietary exposure of glyoxal and methylglyoxal in cookies and their relationship with other heat-induced contaminants. J. Agric. Food Chem..

